# Investigation of wound healing and anti-inflammatory activity of Senna occidentalis leaf extract, and in silico screening for both activities

**DOI:** 10.1016/j.pscia.2023.100016

**Published:** 2023-10-14

**Authors:** Md.Abu Shyeed, Mahci Al Bashera, Ovijit Sarkar Sazal, Md.Moktar Ali, Md Polok Hossain, Henry Sandip Kumar Mondol, Mohammad Ali Chowdhury, Khan Rajib Hossain, Md Tamzid Hossain Molla

**Affiliations:** a Department of Applied Chemistry and Chemical Engineering, Rajshahi University, Rajshahi, 6205, Bangladesh; b BCSIR Rajshahi Laboratory, Bangladesh Council of Scientific and Industrial Research (BCSIR), Dhaka, 1205, Bangladesh; c Department of Anatomy, Kumudini Women's Medical College, Tangail, 1940, Bangladesh; d Department of Anesthesia, Rajshahi Medical College, Rajshahi, 6000, Bangladesh; e State Key Laboratory of Solid Lubrication, Lanzhou Institute of Chemical Physics, Chinese Academy of Sciences, Lanzhou 730000, China

**Keywords:** Senna occidentalis, Cell proliferation, Membrane stabilization, **Drug likeliness**, **Ligand-protein complex stabilization**

## Abstract

Senna occidentalis, synonym Cassia occidentalis, is a native American pantropical plant species previously classified under the genus Cassia. This study is for testing and to discover new potent phytochemicals from this plant as wound healing and anti-inflammatory agents. The Excision and Assay of Red Blood Cell (RBC) Membrane Stabilization for Anti-Inflammatory Activity Test Method was used to test how well extracts of *S. occidentalis* leaves from methanol, n-hexane, chloroform, and absolute alcohol helped wounds heal and stopped inflammation. In silico **is another method** for finding potent phytochemicals for both activities. These leaf extracts effectively **cure wound areas and promote re-epithelialization**. The methanol extract exhibited maximum wound healing (95.04%) and anti-inflammation (62.94%) activity compared to their other extracts, standard, and control groups. In silico **molecular docking of Apigenin**, Aloe-emodin with GSK-3B protein, and 1-Methoxynaphthalene, Quinine with COX-2 protein showed binding affinity in **(kj**J**/mol)** of −8.4, −8.6, and −7.3, −7.7, for wound healing and anti-inflammatory activity, respectively, in their binding sites with stability. They support the **"Lipinski Rule of Five."** This plant leaf extract is recommended as a traditional medicine and an alternative, complementary treatment for its continued contribution to drug discovery and development.

## 1 Introduction

Senna occidentalis (Indian vernacular name Usaya ki Fali [1]) is a pantropical plant species native to the American, and its synonym is Cassia occidentalis. The species was formerly placed in the genus Cassia. Vernacular names in English include septic weed and coffee senna [2]. And some significant phytochemicals are chrysophanol, aloe emodin, apigenin, 1-methoxynaphthalene, nerolidol, and quinine which have in this plant [3]. Numerous therapeutic uses, including antiparasitic, antibacterial, antifungal, anticancer, anti-inflammatory, and analgesic activities, have been documented for it [4]. In rural and urban settings, *S. occidentalis* has some food applications; steaming green leaves, blossoms, and seeds are used as vegetables, and even the dried crushed seed is used as an alternative to coffee. This plant has enough anthraquinones, flavonoids, tannins, alkaloids, steroids, glycosides, phenolics, terpenes, and saponins, and its multiple uses have led us to forward it for further therapeutic activity testing [5]. Traditional medicine has extensively documented the therapeutic benefits of medicinal plants, which have been used for ages to cure various ailments [6,7]. Scientists can find active substances that could be used to create new medications by researching these plants' wound healing, anti-inflammatory, antidiabetic, anticancer, hepatoprotective, hypolipidemic, antimalaria, antioxidant, and antibacterial properties. Besides drug discovery, other focuses of these biological activity tests are reduced side effects, cost-effective treatment, increased understanding of natural remedies, and addressing antibiotic resistance. A significant public health issue is antibiotic resistance. Scientists can find new compounds that could be utilized to fight antibiotic-resistant bacteria by researching the antibacterial properties of medicinal plants [8].

Numerous illnesses and ailments have been researched concerning the Senna occidentalis plant. Following are some references to studies that looked into how Senna occidentalis affected certain diseases: Antibacterial [9], wound healing [10], antidiabetic [11], anticancer [12], hepatoprotective [13], hypolipidemic [14], antimalarial [15], anthelmintic [16], antioxidant and anti-atherosclerogenic [17], and anti-inflammatory [18] are some of the other properties listed. Senna occidentalis (Cassia occidentalis) is a medicinal plant traditionally used for wound healing, anti-inflammatory and antimicrobial properties. The wound-healing activity of Senna occidentalis has been investigated both in vivo and in silico, including its interaction with the Glycogen synthase kinase-3 beta (GSK-3B) protein. Sheeba, M. et al. [19] have studied the therapeutic activity of Cassia occidentalis (Senna occidentalis) leaf extract for wound healing, and their results were supportive. Also, the anti-inflammatory activity of Senna occidentalis has been investigated both in vitro and silico, including its interaction with the cyclooxygenase-2 (Cox-2) protein [20]. Inflammation is another growing disease, and Basha, Syed Ilias et al. have proved that *C. occidentalis* (Senna occidentalis) seed extract can be used as an anti-inflammatory agent [21]. Recent studies have investigated the plant's antimicrobial activity and have found that it has the potential to inhibit the growth of a wide range of microorganisms, including bacteria, fungi, and viruses [22]. Arya Vedpriya et al., in their study, illustrated that Cassia occidentalis leaf extract effectively kills bacteria [23]. Furthermore, Mohammed M et al. [24] showed that leaf extract is a potent antimicrobial agent. In vivo, studies have demonstrated that Senna occidentalis leaf extract can promote the healing of wounds by increasing the rate of wound closure, reducing wound size, and enhancing the formation of new blood vessels. These effects may be due to the plant's ability to stimulate collagen synthesis, strengthen fibroblast proliferation, and modulate the inflammatory response. In vitro Senna occidentalis extracts exhibit anti-inflammatory activity by inhibiting the production of pro-inflammatory cytokines such as interleukin-1β and tumor necrosis factor-alpha [25]. These effects may be due to the plant's ability to modulate the activity of enzymes involved in inflammation, such as cyclooxygenase-2 (COX-2) and lipoxygenase (LOX) [26].

Computational studies have explored the potential mechanisms behind Senna occidentalis' wound healing and anti-inflammatory activity, including its interaction with the GSK-3B and Cox-2 receptor proteins for wound healing and anti-inflammatory activity. GSK-3B is a criticalprotein that regulates various cellular processes, including wound healing [19,21]. In one study, molecular docking simulations were performed to investigate the interactions between Senna occidentalis compounds and the GSK-3B protein. The results showed that the plant's bioactive compounds could bind to and inhibit the activity of GSK-3B, which may contribute to the plant's wound-healing activity. COX-2 is an enzyme involved in the production of prostaglandins, which play a role in the inflammatory response [27]. Molecular docking and simulations were performed to investigate the interactions between Senna occidentalis compounds and the Cox-2 protein. Evidence proved that the plant's bioactive compounds could bind to and inhibit the activity of Cox-2, which may contribute to the plant's anti-inflammatory activity. Studies suggest Senna occidentalis possesses wound healing, anti-inflammatory, and antimicrobial activity. Due to its ability to stimulate collagen synthesis, enhance fibroblast proliferation, modulate the inflammatory response, and inhibit the activity of essential proteins such as GSK-3B for wound healing. Ability to modulate the activity of enzymes involved in inflammation, such as COX-2 and LOX, for anti-inflammatory activity. Further research is needed to fully understand the mechanisms of action and explore Senna occidentalism's potential as a therapeutic agent for wound healing and anti-inflammatory and antimicrobial activity [28]. In this study, leaf leaves extract of *S. occidentalis* has been forwarded to investigate wound healing and anti-inflammatory activity.

In this study, the leaf extract of *S. occidentalis* has been forwarded to investigate wound healing and anti-inflammatory activities and to find out some phytochemicals that are biocompatible and active agents to remedy illness. According to this research, several phytochemicals were selected for in silico screening and found to have the desired result of being capable of activating wound and inflammation recovery. Apigenin and aloe-emodin showed better therapeutic activity for wound healing, whereas 1-methoxy naphthalene and quinine were potential phytochemicals against inflammation. Senna occidentalis is a potential source of these phytochemicals, and it can be cultivated in the local area to make it available everywhere. As day-by-day medicine is going to be resistant to specific diseases, drug development is an indispensable matter, and the purpose of this study was to discover some new phytochemicals from this plant that can play a role in drug development.

## 2 Method and materials

### 2.1 Plant extraction

Selected Senna Occidentalis (Kalkasunda) leaves were collected from the Rajshahi University, Bangladesh, campus botanical garden in June and July. The leaves were air-dried (26​°C), then powdered and prepared for solvent extraction (hot extraction). Some physical tests were done on the leaves, such as the percentage of moisture content, ash content, and dry matter content of the plant leaves. Finally, leaves were extracted with N-hexane, chloroform, methanol, and absolute alcohol, where the leaves’ powders were reused from one solvent to another. In that case, the powder materials were dried after extraction with one solvent to prepare it for another extraction with different solvents using a soxhlet extraction; moreover, solvent and extract mixtures were distilled to separate the solvent and extracted material. The isolated extract material was dried at room temperature for further progress. The extraction of leaves powder in the Soxhlet apparatus was carried out at the boiling points of these four solvents, i.e., N-Hexane (68.7​°C), Chloroform (61.2​°C), Methanol (64.7​°C), and Absolute alcohol (78.37​°C). In the case of extraction, every solvent quantity was 800 ​ml, and the leaves powder was 100 ​± ​5 g. Every cycle in the extraction took 12 ​min and approximately 6 ​h for 30 cycles for each solvent extraction. Hot extraction (soxhlet extraction) includes heating the plant material or solvent to make the desired chemicals more soluble and enable the extraction of more compounds in less time. On the other hand, cold extraction frequently takes longer than hot extraction and may not produce a fully extracted product [29].

### 2.2 Preparation of ointment

To make extract ointment using an ointment base (Vaseline) [30], four types of extracts (n-hexane extract, chloroform extract, methanol extract, and absolute alcohol extract) were used. Each section weighed at 900 ​mg and was added to a beaker containing 10 ​ml of melted ointment base. The mixture was then vigorously mixed to create a homogenous mixture. Then it was stored at room temperature to observe phase separation, color, odor, and stability.

### 2.3 Experimental animal

54 Swiss albino mice (weighing 25–30 g) were collected from the Zoology Department, Rajshahi University, Bangladesh. They were provided with a polypropylene cage, a balanced diet, and free access to water. The mice's health conditions were checked daily, and they rested for 14 days before the experiment. The suitable temperature of 200–250 °C, relative humidity of 45–65%, and dark light cycle of 12 ​h were maintained during the experiment [31].

### 2.4 Wound creation

**24** healthy mice were taken for wound creation, and food was not provided 12 ​h before the mice received proper anesthesia using ketamine. The mice's dorsal parts were saved one by one using an electric trimmer, and the wound was created by sterilized forceps and a surgical blade [32]. The method was excision. Before wound creation, the specified area was marked with a marker pen to create the desired wound.

### 2.5 Blood collection

Thirty healthy mice who did not receive NSAIDs two weeks before the experiment was selected. 15 ​ml of blood was collected from 30 mice tail cords using a 3 ​ml syringe and needle; before collecting the blood, the tails were touched with isopropyl alcohol [33]. The collected blood was kept in a test tube containing isotonic solution and ethylenediamine tetraacetic acid (EDTA) [34], which acts as an anticoagulant, before the centrifuge began.

### 2.6 Preparation of 10% RBC suspension

The collected blood was centrifuged at 3000 ​rpm for 20 ​min, and the supernatant was removed to separate packed red cells. Then it was washed with normal saline, and the total process was repeated 4 times. According to (Oyedapo et al., 2004), 10% RBC suspension was prepared by mixing 50 ​μL of RBC in 2 ​ml of normal saline [33].

### 2.7 Experiment design for wound healing

Twenty-four wounded mice were divided into 6 groups after the creation wound. Each group contains 4 mice. Groups I, II, III, and IV were tested; group V was standard, and VI was the control group. Prepared ointment of extracts, ointment base (vaseline), and standard were applied on the wound of mice using sterilized swabs. These ointments were laid on the wounded surface of the mice carefully to give proper protective film on the wounded surface. They kept observation at the time of treatment to avoid any types of unwanted occurrence. The recovery wounded area was measured from time to time to get proper recovery information, and the treatment duration was 10 days. Group I: methanol extract 900 mg/10 ​ml, Group II: n-hexane extract 900 mg/10 ​ml, Group III: chloroform extract 900 mg/10 ​ml, Group IV: Absolute alcohol extract 900 mg/10 ​ml, Group V: Silver sulphadiazine. (standard), Group VI: ointment base (control), Percentage of healing can be calculated using the following formula [35].

$$\%Wound\;Healing\;Percentage=\frac{\left(\text{Wound}\;\text{area}\;\text{at}\;0\;\text{day}\;–\;\text{Wound}\;\text{area}\;\text{at}\;\text{nth}\;\text{day}\right)}{\text{Wound}\;\text{area}\;\text{at}\;0\;\text{day}}\;X100$$

### 2.8 Assay of Red Blood Cell (RBC) Membrane Stabilization for Anti-Inflammatory Activity Test

To evaluate the anti-inflammatory activity of the *S. occidentalis* leaves extract RBC membrane stabilization technique was incorporated by following Gandhidasan et al. [36] Methanol, N-hexane, and chloroform extract were used as the test groups, whereas indomethacin was used as the standard drug. The percentage of RBC-lysis, including lysosomal membrane, indicates the efficient anti-inflammatory activity of the drug. The following samples were prepared to investigate the anti-inflammatory activity of the extracts. In the mixture (control) of 2 ​​ml of hypotonic saline (0.25%, w/v), 1 ​ml of phosphate buffer (pH7.4), and 0.5 ​ml of 10​% w/v mouse red blood suspension for the test solution, 1 ​ml of test extract (5 ​mg/ml – 8 ​mg/ml) was mixed. 1 ​ml of Indomethacin (2.5 ​mg/ml) was mixed with the standard solution. Before centrifugation at 3000 ​rpm for 20 ​min, the solution was incubated for 30 ​min at 37°C. Then, the supernatant absorbance was measured using a spectrophotometer at 560 ​nm. The percentage inhibition of haemolysis or membrane stabilization was calculated using the following equation [37,38].

$$\%\;Inhibition\;of\;haemolysis=\frac{A_{1}-A_{2}}{A_{1}}\;X100$$

Where: A_1_ ​= ​Absorption of hypotonic buffered saline solution alone; A_2_ ​= ​Absorption of test sample in hypotonic solution.

In Silico Screening for Wound Healing and Anti-Inflammatory Activity Investigation of *S. occidentalis* Leaf.

### 2.9 Receptor protein and ligand preparation

60 phytochemicals of **S. occidentalis** leaves were taken from the phytochemical databank IMPPAT as 3D SDF. The receptor Protein GSK3-β (PDB: 5K5N) [19] and COX- 2 (PDB: 6COX) [21] were downloaded from the protein data bank (PDB), respectively, for wound healing and anti-inflammatory activity. The raw protein, which contained water molecules, foreign metal ions, heavy atoms, and cofactor, was cleaned by Pymol software.

### 2.10 Molecular docking

60 phytochemicals were assessed to observe the therapeutic activity against wound and inflammation through the binding affinity with the receptor proteins using auto dock vina in Pyrx software—the grid box provided this data, which showed in Table 1.

**Table 1:** Grid box data for GSK-3B, COX-2 and Ligand.

Grid box data for GSK-3B and Ligand		Grid box data for COX-2 and Ligand	
Centre (Å)	Dimension (Å)	Centre (Å)	Dimension (Å)
X ​= ​10.7476	X ​= ​69.1414	X ​= ​27.7710	X ​= ​58.61
Y ​= ​1.2002	Y ​= ​50.2062	Y ​= ​29.3794	Y ​= ​74.8526
Z ​= ​28.3750	Z ​= ​58.8523	Z ​= ​40.1340	Z ​= ​63.2432

### 2.11 Drug likeliness

Phytochemicals are naturally occurring plant substances that may have therapeutic benefits. The likelihood that a molecule will be an orally accessible medicine with suitable pharmacokinetic and pharmacodynamic features is known as drug likeliness. This idea is crucial for drug discovery since it can help create effective medications by discovering molecules with a high drug likeliness. The molecular weight, lipophilicity, hydrogen bond donors, acceptors, and topological polar surface area of a compound are some variables that affect its likelihood of being a medication. Compared to molecules outside this range, those with molecular weights between 250 and 500 ​Da are more likely to be bioavailable orally.

Moreover, the quantity of hydrogen bond donors and acceptors might affect the likelihood that a medicine will be effective, with an excess of either reducing the likelihood of oral bioavailability. The surface area of a molecule that is available for generating polar interactions is another essential component in predicting a drug's possibility of being effective. The top 12 phytochemicals (6 phytochemicals for wound healing and 6 phytochemicals for anti-inflammatory activity) with the highest binding affinity from the pkCSM (predicting small-molecule pharmacokinetic properties using graph-based signatures) database have had their ADMET examined in this study [39].

### 2.12 Molecular dynamic (MD) simulation

A powerful computer method used to examine the behavior of molecules over time is molecular dynamics (MD) simulation. Desmond is a well-known piece of software that D. E. Shaw Research created for running MD simulations. Researchers must define parameters such as force fields and simulation conditions, to run MD simulations using Desmond. The intermolecular interactions between atoms in a molecular system are modeled mathematically using force fields. Desmond employs OPLS-3e force fields to simulate a variety of biological systems precisely. The type of system being researched and the particular research topic being addressed influence the force field selection [40]. The simulation time step, temperature (300 k), pressure (1.63 ​bar), and boundary conditions are other crucial elements in MD simulation [41]. These parameters must be carefully selected to provide accurate and trustworthy simulation results. For instance, the time step must be small enough to represent the system's dynamics accurately, and the temperature and pressure must be regulated to closely resemble the biological environment's circumstances. Desmond offers several tools for setting and altering simulation settings and analyzing and displaying simulation results [42]. This enables scientists to develop a thorough grasp of the behavior of their systems and to plan wisely for upcoming studies.

## 3 Results

The dried plant was appropriately stored at room temperature; the percentages of the plant leaves' moisture content, ash content, and dry matter content were 11.34​%, 14.68​%, and 88.66​%, respectively. The prepared extract-ointment color, odor, and physical state were stable at room temperature. All the experimental animals were healthy before the experiment at a temperature of 20–25​°C, relative humidity of 45–65​%, and a dark light cycle of 12 ​h, which was maintained during the experiment period, and no infection was observed in the wound-created mice. The blood collected from mice did not clot due to the isotonic solution and ethylenediamine tetraacetic acid (EDTA). Even the bacterial colony formation was satisfactory.

### 3.1 Result for wound healing activity test

**Fig. 1:**
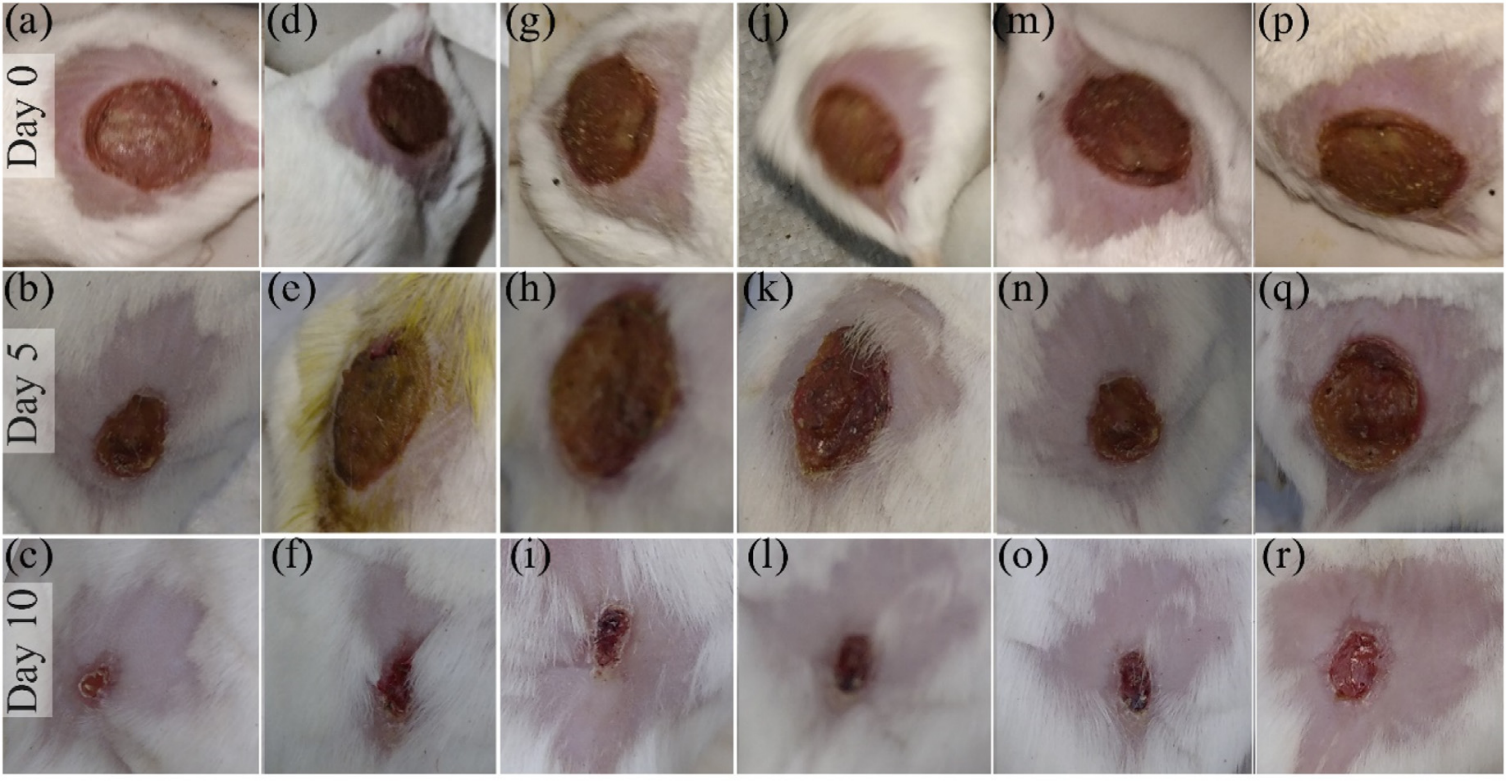
Images showing day 0, 5, and 10 healing progress of the (a–c) Methanol extract, (d–f) N-Hexane extract, (g–i) Chloroform extract, (j–l) Absolute alcohol extract, (m–o) Standard, and (p–r) Control.

**During the** treatment of tested animals, after every 5 days, wound recovery data (Table 2) were taken to compare the therapeutic activity of different extract ointments with a standard. On the 5th day, methanol extract-ointment exhibited a maximum healing area of 28.13​% and control 12.6​%. On the 10th day, methanol extract-ointment and control showed healing activity at 95.04​% (maximum) and 47.19%, respectively. Standard exhibited 26.19% and 87.31​% healing activity on the 5th and 10th days, respectively, to which other extract activities have to compare. The percentage of wound healing for all groups is given in Table 3. Treatment time from day 0 to day 10 is given in Fig. 1, and the healing activity of the wounded surface of mice is shown in the Table 2 below in mm^2^.

**Table 2:** Wounded surface area at 0 to 10th day.

Day	Group of Injured surface area in mm^2^					
	I	II	III	IV	V	VI
0	143.14	213.3	127.67	132.73	122.72	172.312
5	102.87	160.22	99.18	104.76	90.58	150.6
10	7.09	12.56	9.621	9.621	15.57	90.99

**Table −3:** Percentage of wound healing.

Day	Group					
	I	II	III	IV	V	VI
**5**	28.13 ​%	24.88 ​%	22.31 ​%	21.07 ​%	26.19 ​%	12.60 ​%
**10**	95.04 ​%	94.11 ​%	92.46 ​%	92.75 ​%	87.31 ​%	47.19 ​%

### 3.2 Result for anti-inflammatory activity by RBC membrane stabilization

The therapeutic activity of extracts against inflammation showed a maximum percentage of inhibition of haemolysis in methanol extract (8 ​mg/ml) 62.94% and minimum in Chloroform extract (5 ​mg/ml) 16.24%. Indomethacin (standard) showed a percentage of inhibition of haemolysis 76.14%. All the results are shown in below Table 4 and graphically in Fig. 6, % of inhibition (in the discussion section).

**Table 4:** Percentage of inhibition of haemolysis.

Treatment	Concentration (mg/ml)	Absorbance (560 ​nm)	% of Inhibition
Methanol Extract	5	0.098	50.25
	6	0.087	55.83
	7	0.079	59.89
	8	0.073	62.94
N-hexane Extract	5	0.134	31.97
	6	0.127	35.53
	7	0.123	37.56
	8	0.120	39.08
Chloroform Extract	5	0.165	16.24
	6	0.153	22.34
	7	0.150	23.85
	8	0.142	27.91
Indomethacin	2.5	0.047	76.14
Control		0.197	

## 4 Result for *In silico* screening

### 4.1 Receptor protein preparation

**Fig. 2:**
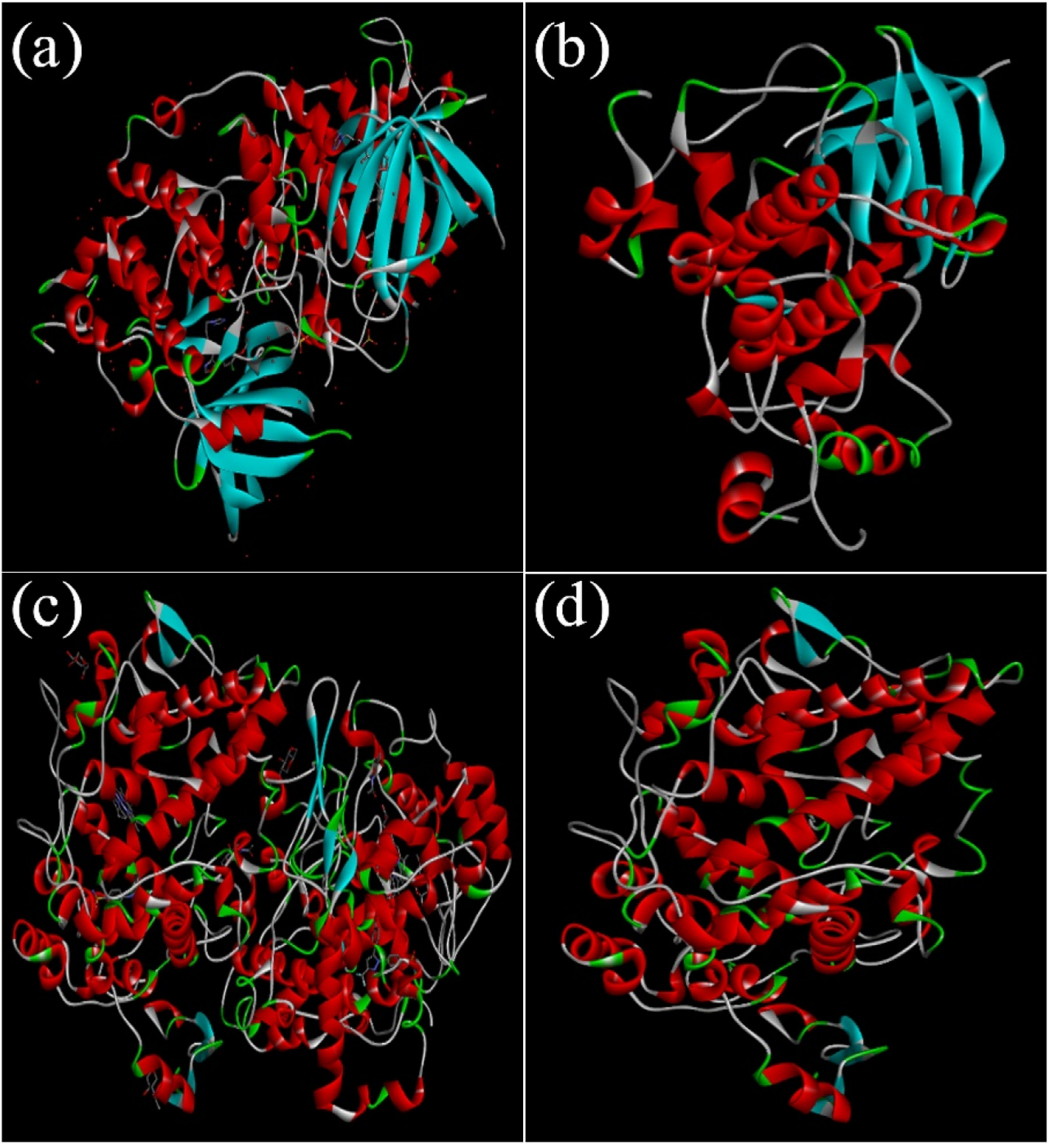
Illustration of the (a) retrived protein from RCSB protein databank (GSK-3B), (b) protein after cleaning, (c) retrived protein from RCSB protein databank (COX-2), and (d) protein after cleaning.

All water molecules, ligands, and other hetero atoms were eliminated from the structures to prepare the protein. The atoms' valencies were filled with hydrogen atoms. It has been illustrated in Fig. 2.

### 4.2 Molecular docking studies

All phytochemicals with COX-2 binding energy ranged from −9.1 ​kJ/mol to −6.5 ​kJ/mol for anti-inflammatory efficacy, and the top 6 phytochemicals were chosen based on binding affinity, which ranged from −9.1 ​kJ/ml to 6.5 ​kJ/ml. The top binding affinities of the six phytochemicals were chosen for wound healing and ranged from −8.8 kJ/mol to −7.7 kJ/mol for phytochemicals with GSK-3B. These all are shown in Table 5 below data from pyRx.Where, A ​= ​Chrysophanol; B= Aloe emodin; C ​= ​Apigenin; D**=**1-Methoxynaphthalene; E ​= ​Nerolidol; F ​= ​Quinine.

**Table 5:** Binding affinity (kj/mol) between ligands and receptor protein (GSK-3B and COX-2).

Ligand	A	B	C	D	E	F
Binding Affinity (kj/mol) Between Ligand and GSK-3B Protein	−8.7	−8.6	−8.4	−7.7	−7.7	−7.9
Binding Affinity (kj/mol) Between Ligand and COX-2 Protein	−9.1	−7.1	−6.5	−7.3	−6.9	−7.7

### 4.3 Drug likeliness properties

According to the binding affinity, the drug likeliness of 6 selected phytochemicals was analyzed for wound healing and anti-inflammatory activities. These data were collected from the Swiss ADME database. All data are mentioned below in Table 6. Where, A ​= ​Chrysophanol; B= Aloe emodin; C ​= ​Apigenin; D**=**1-Methoxynaphthalene; E ​= ​Nerolidol; F ​= ​Quinine.

**Table 6:** Drug likeliness properties of selected ligands.

Ligand		A	B	C	D	E	F
Physicochemical Properties	Molecular weight	254.24 ​g/mol	270.24 ​g/mol	270.24 ​g/mol	158.20 ​g/mol	222.37 ​g/mol	324.42 ​g/mol
	Num. heavy Atoms	19	20	20	12	16	24
	Num. rotatable Bonds	0	1	1	1	7	4
	Num. H-bond Acceptors	4	5	5	1	1	4
	Num. H-bond Donors	2	3	3	0	1	1
Lipophilicity	LogP	2.18162	1.3655	2.5768	2.8484	4.3963	3.1732
Druglikeness	Violation	0	0	0	0	0	0

### 4.4 Molecular interaction analysis

**Fig. 3:**
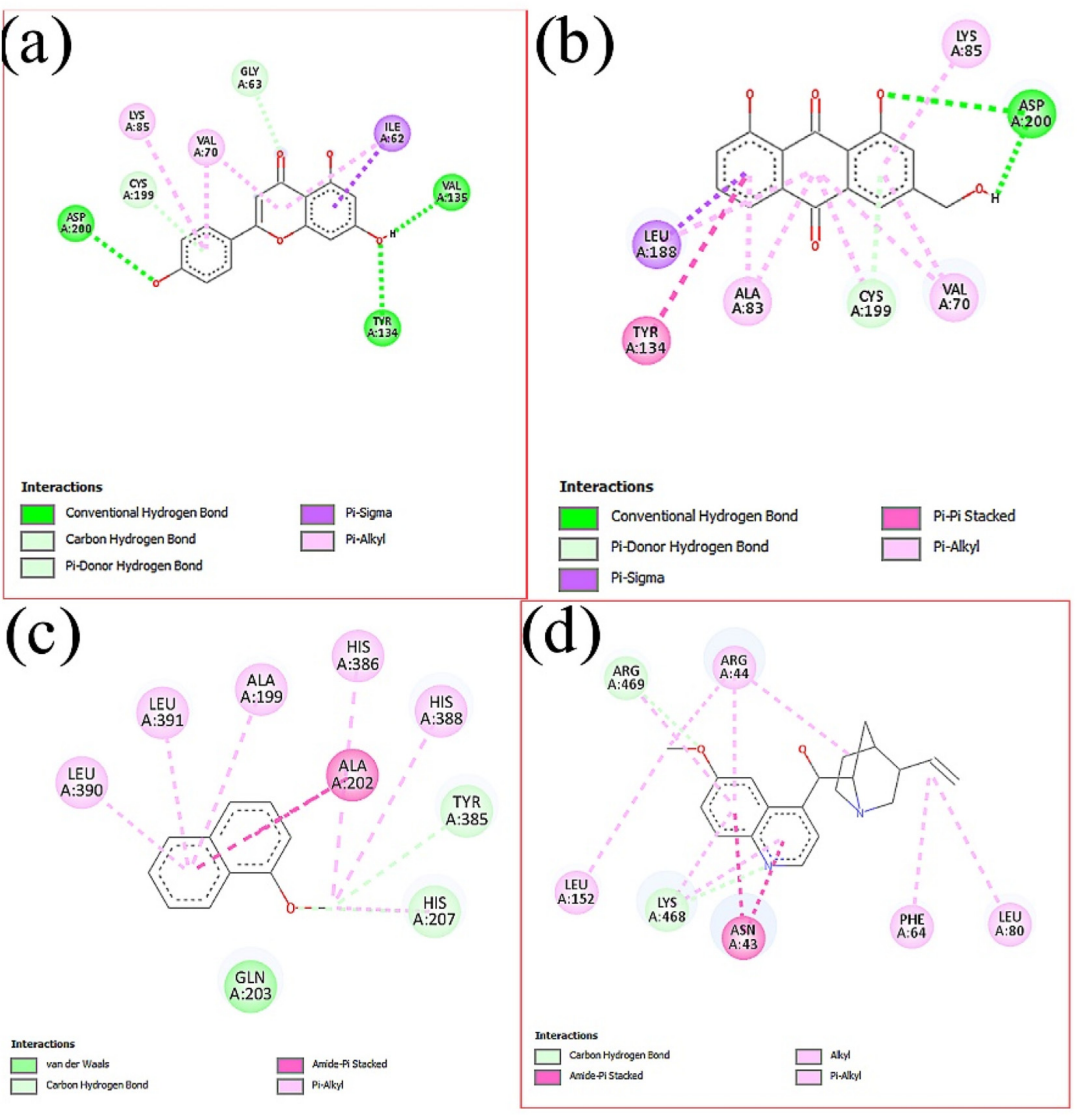
Image of the molecular (a) Interaction between Apigenin and GSK-3B, (b) Interaction between Aloe emodin and GSK-3B, (c) Interaction between 1-Methoxynaphthalene and COX-2, and (d) Interaction between 1-Methoxynaphthalene and COX-2.

After analysis of the binding affinity and drug-likeliness properties, two phytochemicals were selected to observe the molecular interaction between a ligand and the receptor protein GSK-3B for wound healing. Two more phytochemicals with the COX-2 receptor protein are being examined for their ability to reduce inflammation in the Discovery Studio. These are illustrated in Fig. 3. Moreover, interactions with the binding site are also shown in Table 7.

**Table −7:** Interactions with the binding site of Ligands and Receptor protein (GSK-3B and COX-2).

Interaction between Apigenin and GSK-3B	ASP A:200, TYR A: 134, VAL A:135, CSY A:199, LSY A:85, VAL A:70, GLY A:63 and ILE A:62
Interaction between Aloe emodin and GSK-3B	LEU A:188, TYR A:134, ALA A:83, CYS A:199, VAL A:70, ASP A:200 and LYS A:85
Interaction between 1- Methoxynaphthalene and COX-2	LEU A:390, LEU A:391, ALA A:199, HIS A:386, HIS A:388, ALA A:202, TYR A:385, HIS A:207 and GLN A:203
Interaction between Quinine and COX-2	LEU A:152, LYS A:468, ASN A:43, PHE A:64, LEU A:80, ARG A: 44 and ARG A:469

### 4.5 Molecular dynamic simulation

**Fig. 4:**
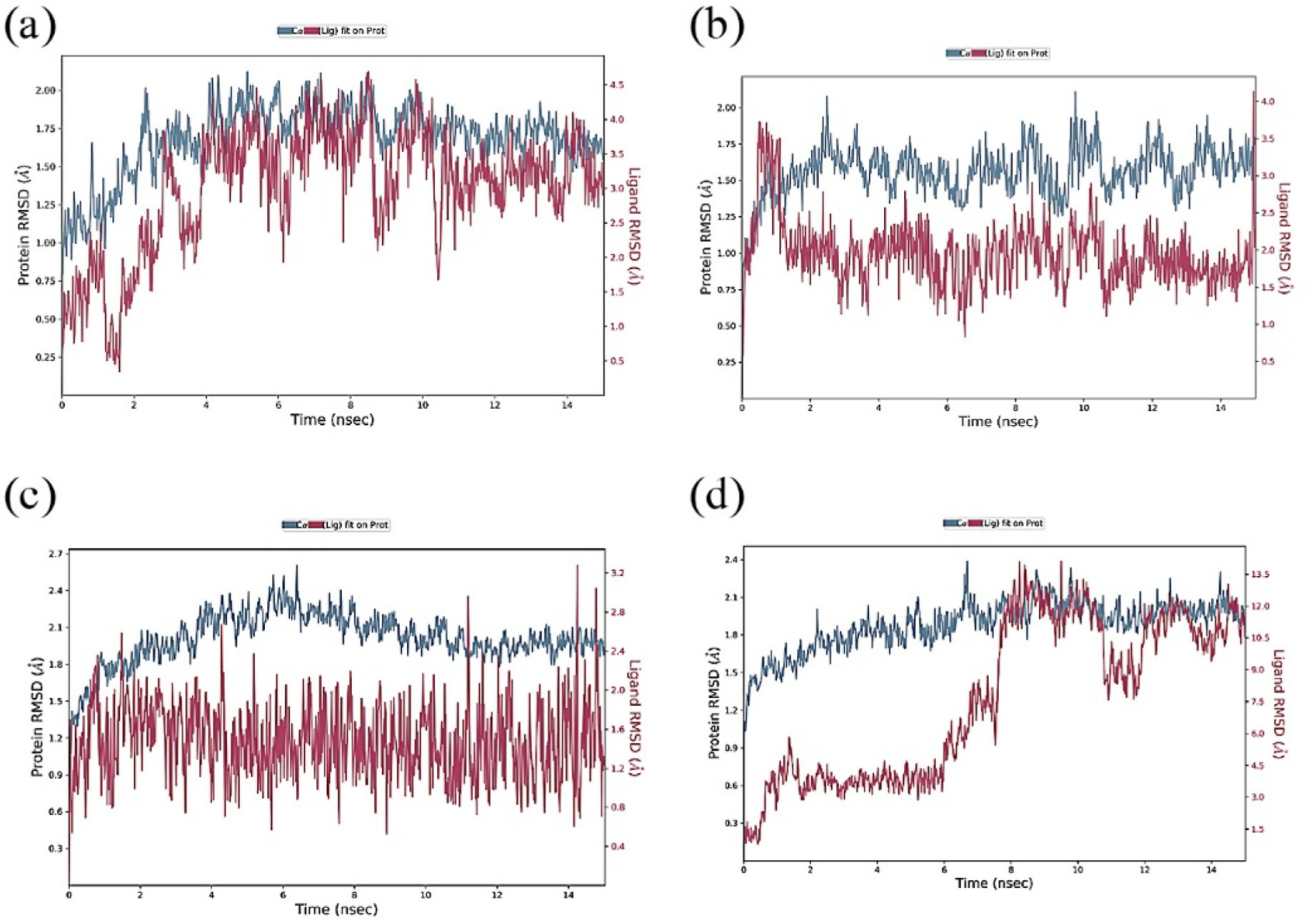
Graphical view of the (a) RMSD between Apigenin and GSK-3B, (b) RMSD between Aloe emodin and GSK-3B, (c) RMSD between 1-Methoxynaphthalene and COX-2, and (d) RMSD between Quinine and COX-2.

In a ligand-protein complex, MD simulation is used to understand the systems's dynamic behavior, including the thermodynamics, kinetics, and structural alterations that occur during binding. The protein RMSD acceptable value, ranging from 1 ​Å to 3 ​Å, can offer crucial details regarding the structural modifications in the protein-ligand complex that occur throughout the simulation. RMSD plots typically display the RMSD values as a function simulation time, enabling viewing of any substantial alterations to the complex's structure, as shown in Fig. 4. The protein-ligand complex is stable, and the structure is preserved across the simulation time if the RMSD value is low. A high RMSD value, on the other hand, denotes the instability of the protein-ligand combination and suggests that the ligand may be moving away from the receptor. It's vital to remember that to learn more about how proteins interact with ligands during the simulation, the RMSD value should be studied in conjunction with additional metrics, such as RMSF (Root Mean Square Fluctuation) or H-bond analysis [43]. The ligands (Aloe-emodin, Apigenin, 1- Methoxynaphthalene, and Quinine) and receptor protein RMSD plots have given in Fig. 4.

### 4.6 Discussion for wound healing

Inflammation, cell proliferation, and tissue remodeling are just a few of the physiological processes that play a role in the complicated wound-healing process. Plant extracts are among the natural items whose potential for wound healing has been investigated. The most widely used solvents for extracting plant bioactive chemicals include methanol, n-hexane, chloroform, and pure alcohol. Here, we'll discuss how mice heal wounds using extracts from methanol, n-hexane, chloroform, and absolute alcohol. According to studies, methanol extract accelerates collagen production, boosts angiogenesis, and lowers inflammation in mice wounds. By stimulating wound contraction and epithelialization, it facilitates wound healing in mice. This is attributed to the increased activity of growth factors and the stimulation of collagen synthesis.

**Fig. 5:**
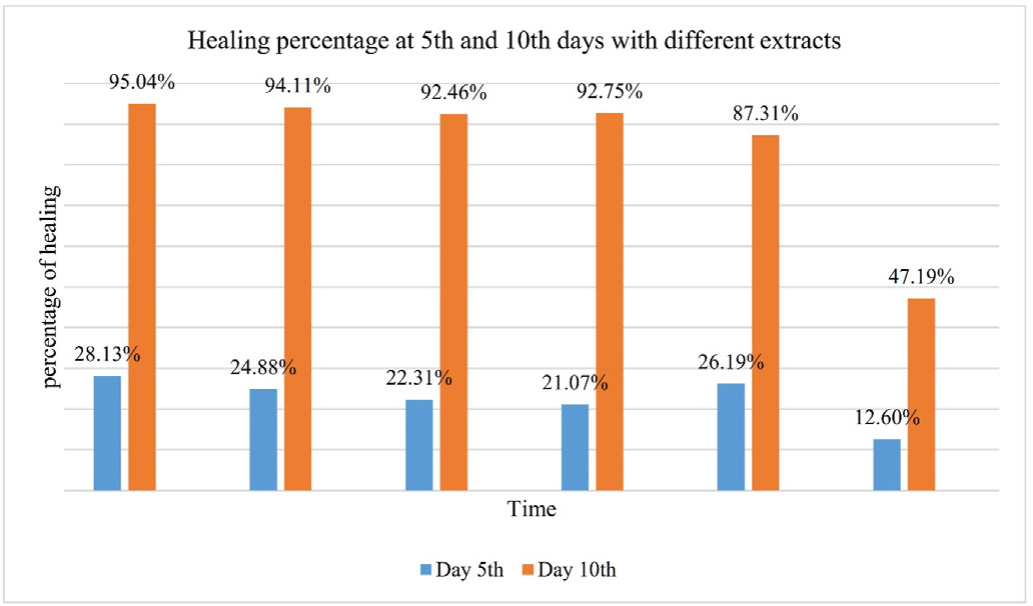
Healing percentage at 5th and 10th days with different extracts.

Moreover, it has been discovered that extracts of N- hexane, chloroform, and absolute alcohol help mice's wounds heal [44]. Sequentially, the maximum percentage of healing was exhibited in groups I (methanol extract - 95.04​%), group-II (n-hexane extract −94.11​%), group-IV (absolute alcohol extract −92.75​%), and group-III (chloroform extract −92.46​%) compared to group-V (standard −87.31​%) and group-VI (control −47.19​%) illustrated in Fig. 5. The plant extract has been shown to quicken the healing of wounds in mice by boosting collagen production and fibroblast proliferation. The extract's flavonoids, polyphenols, and tannins, known to encourage tissue regeneration, were said to be responsible for this impact. In conclusion, it has been discovered that the methanol, ethanol, n-hexane, and chloroform extracts of different plants can speed up the healing of wounds in mice by promoting collagen synthesis, boosting angiogenesis, and lowering inflammatory response [45]. The presence of bioactive substances in the extracts, including flavonoids, tannins, and polyphenols, is thought to be responsible for these effects. More research is required to determine the active compounds in mice that perform these tasks and their mode of action. Before using these extracts in clinical settings, assessing their efficacy and safety is crucial.

### 4.7 Discussion for anti-inflammatory activity

**Fig. 6:**
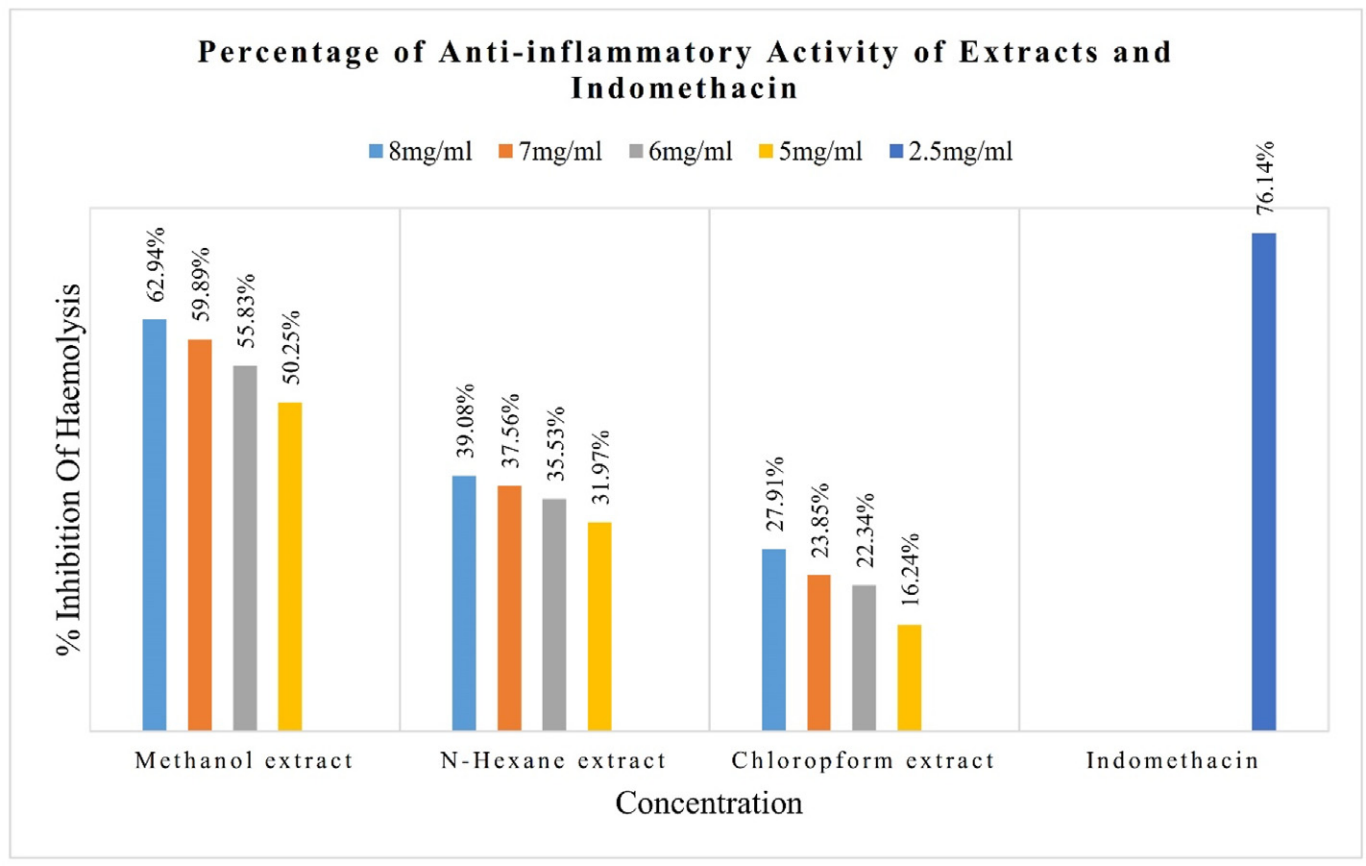
Percentage of anti-inflammatory activity of extracts.

"Lysosomal hydrolytic enzymes" refers to enzymes commonly found in lysosomes. These membrane-bound organelles are present in the majority of eukaryotic cells. Proteins, carbohydrates, and nucleic acids are just a few examples of the different biological substances these enzymes are in charge of dissolving into smaller parts that the organism may utilize. An inflammatory response is a sophisticated natural reaction to adverse stimuli like germs, damaged cells, or irritants. Lysosomal hydrolytic enzymes from immune cells' lysosomes can be discharged into the surrounding tissue in certain types of inflammation, such as chronic inflammation [46]. Cell death, lysosomal rupture, or active secretion by immune cells are just a few of the mechanisms that can cause this release. Lysosomal hydrolytic enzymes can damage extracellular matrix proteins and other tissue constituents; therefore, their release into the extracellular space can be detrimental to the tissue around it. This deterioration can cause tissue damage and speed up the development of several inflammatory disorders, including atherosclerosis and rheumatoid arthritis. Red blood cells (RBCs) are crucial for preserving the microcirculation and oxygenation of the tissues, and their malfunction can lead to inflammation and tissue damage. Lysosomal hydrolytic enzymes can reduce inflammation by improving RBC membrane stability by combining medicinal plant extracts with various solvents, including methanol, n-hexane, and chloroform. It has been demonstrated that several medicinal herbs contain anti-inflammatory and RBC membrane-stabilizing effects. For instance, it has been shown that extracts from plants like *S. occidentalis*, Ocimum sanctum, and Terminalia arjuna can prevent oxidative damage to RBC membranes and limit the activity of lysosomal hydrolytic enzymes [33]. *S. occidentalis* plant leaves are extracted with methanol, n-hexane, absolute alcohol, and chloroform, which contain various bioactive components with antioxidant and anti-inflammatory effects, such as flavonoids, triterpenoids, and phenolic compounds. These substances can neutralize free radicals and stop the action of enzymes like Matrix metalloproteinases (MMPs), which can deteriorate RBC membranes and cause inflammation. These plant extracts have been demonstrated to improve RBC deformability and microcirculation in addition to their anti-inflammatory characteristics, which can further lessen inflammation and tissue damage because these extracts have numerous phytochemicals. The experimentally found result showed statistically below in Fig. 6. Methanol extract is more effective as an anti-inflammatory agent thanother extracts, and methanol extract has therapeutic activity close to indomethacin (standard) [33].

### 4.8 Discussion for in silico screening

Twelve phytochemicals were proposed for molecular interaction and dynamic modeling based on their affinity for binding to GSK-3B and COX-2 proteins. The Lipinski Rule of Five, sometimes referred to as the "Rule of Five," is a series of principles used to assess the drug-likeness of a molecule, and it was used to study and analyze six phytochemicals among them. According to the Lipinski Rule of Five, if a molecule meets the following requirements—MW < 500, Hydrogen bond donors <5, Hydrogen bond acceptors <10, and LogP <5, it is more likely to have oral solid bioavailability and permeability. These guidelines are justified because molecules adhering to them are more likely to exhibit advantageous pharmacokinetic characteristics, such as absorption, distribution, metabolism, and excretion (ADME), in the human body [39]. Drug efficacy may suffer if molecules that defy these regulations have low bioavailability [43]. Aloe-emodin, Apigenin**,** 1-Methoxynaphthalene, and Quinine exhibit acceptable values of the Lipinski rule of five. Protein RMSD values of Apigenin and Aloe-emodin with GSK-3B protein satisfy the RMSD standard value (below 3 ​Å) for wound healing. Furthermore, for anti-inflammatory activity, the protein RMSD value between ligands **(**1- Methoxynaphthalene and Quinine**)** and COX-2 protein is satisfactory and is below 3 ​Å. Interactions between Apigenin and GSK-3B in the active sites are ASP A:200, TYR A: 134, VAL A:135, CSY A:199, LSY A:85, VAL A:70, GLY A:63, and ILE A:62. Interactions between Aloe-emodin and GSK-3B in the active sites are LEU A:188, TYR A:134, ALA A:83, CYS A:199, VAL A:70, ASP A:200, and LYS A:85 for wound healing. Interactions between **1-**Methoxynaphthalene and COX-2 in the active sites are LEU A:390, LEU A:391, ALA A:199, HIS A:386, HIS A:388, ALA A:202, TYR A:385, HIS A:207, and GLN A:203. Interactions between Quinine and COX-2 in the active sites are LEU A:152, LYS A:468, ASN A:43, PHE A:64, LEU A:80, ARG A: 44, and ARG A:469 for anti-inflammatory. Analyzing these, it can be said that *S. occidentalis* leaves extract, which includes these phytochemicals, has therapeutic effects for wound healing and anti-inflammatory activity.

## 5 Conclusion

Senna occidentalis leaf extract's examination of wound healing and anti-inflammatory activity, together with in silico screening for both activities, offers insightful information about the potential therapeutic characteristics of this plant. Through experimental and computational methods, this study attempted to investigate the conventional claims made about the medicinal advantages of *S. occidentalis*. Using mice with excision wounds, the leaf extract's capacity to speed up the healing of wounds was assessed. The outcomes showed that, in comparison to the control group, treatment with the *S. occidentalis* leaf extract considerably expedited wound healing and improved tissue regeneration. The methanol extract was more effective than the standard and control groups at healing the injured mice. The trial results above support the extract's positive effects on wound healing processes such as collagen synthesis, angiogenesis, and re-epithelialization.

In vitro (Assay of Red Blood Cell (RBC) Membrane Stabilization for Anti-Inflammatory Activity Test) models were also used to check if the leaf extract was anti-inflammatory. Compared to other extracts, methanol extract had the highest percentage of hemolysis inhibition during its anti-inflammatory therapeutic effect. This study shows that *S. occidentalis* leaf extract has therapeutic benefits for wound healing in vivo and anti-inflammatory activity in vitro. These benefits are mainly attributable to several phytochemicals in the extract.

To find possible bioactive chemicals found in *S. occidentalis*, in silico screening was carried out using molecular docking and virtual screening approaches. Aloe-emodin, apigenin, 1-methoxynaphthalene, and quinine, which have anti-inflammatory and wound-healing activities, were among the phytochemical compounds discovered. Molecular docking simulations predicted strong binding affinities between these substances and significant molecular targets related to inflammation and wound healing. These studies prove that *S. occidentalis* leaf extract has anti-inflammatory and wound-healing properties. The experimental findings corroborate its potential for therapeutic applications and back up its historical use as a medicine plant. In silico screening provides insightful information about the underlying mechanisms and reveals prospective bioactive substances that could be the source of the observed pharmacological activity. These findings highlight the potential of *S. occidentalis* as a source of new therapeutic agents and add to the body of information on natural compounds with wound healing and anti-inflammatory activities. More research, including clinical trials, is required to investigate the extract's safety, effectiveness, and specific mechanisms of action and its active ingredients.

This study demonstrates the remarkable wound healing and anti-inflammatory properties of *S. occidentalis* leaf extract. The extract has decreased inflammatory markers, including lysosomal hydrolytic enzymes, and enhanced wound closure, tissue regeneration, and epithelialization for wound healing. These actions demonstrate that the plant leaf extract contains bioactive substances. To determine the effectiveness of new chemicals, the association between the chemical makeup of leaf extract and its healing or anti-inflammatory effect is greatly influenced by the quantitative structure-activity relationship (QSAR). Additionally, it can aid in creating new pharmaceuticals and be utilized as conventional medicine as an alternative or complementary treatment for various illnesses.

## Funding information

The author(s) received no financial support for the research, authorship, and/or publication of this article.

## Ethics Approval

This work is approved by the Ethical Review committee of Rajshahi Medical College (RMC/ERC/2021-2022/132/142).

## Credit authorship contribution statement

Md.Abu Shyeed: Writing- Original draft preparation, Methodology, Investigation. Mahci Al Bashera: Conceptualization, Data curation, Visualization. Ovijit Sarkar Sazal, Md. Moktar Ali, Md. Polok Hossain: Writing- Original draft preparation. Henry Sandip Kumar Mondol, Mohammad Ali Chowdhury: Writing- Reviewing and Editing, Animal. Khan Rajib Hossain: Writing- Reviewing and Editing, Software, Investigation. Md. Tamzid Hossain Molla: Supervision,Writing- Reviewing and Editing. All authors have read and approved the final manuscript.

## Data availability

Data will be made available on request.

## Declaration of competing interest

The authors confirm that they have no known financial or interpersonal conflicts that would have appeared to have an impact on the research presented in this study.
