# Supplementary figures and images for: Investigation of wound healing and anti-inflammatory activity of Senna occidentalis leaf extract, and in silico screening for both activities
Source: Pharm Sci Adv. 2023 Oct 14;1(2):100016. doi: 10.1016/j.pscia.2023.100016 (PMC12709893; doi:10.1016/j.pscia.2023.100016)

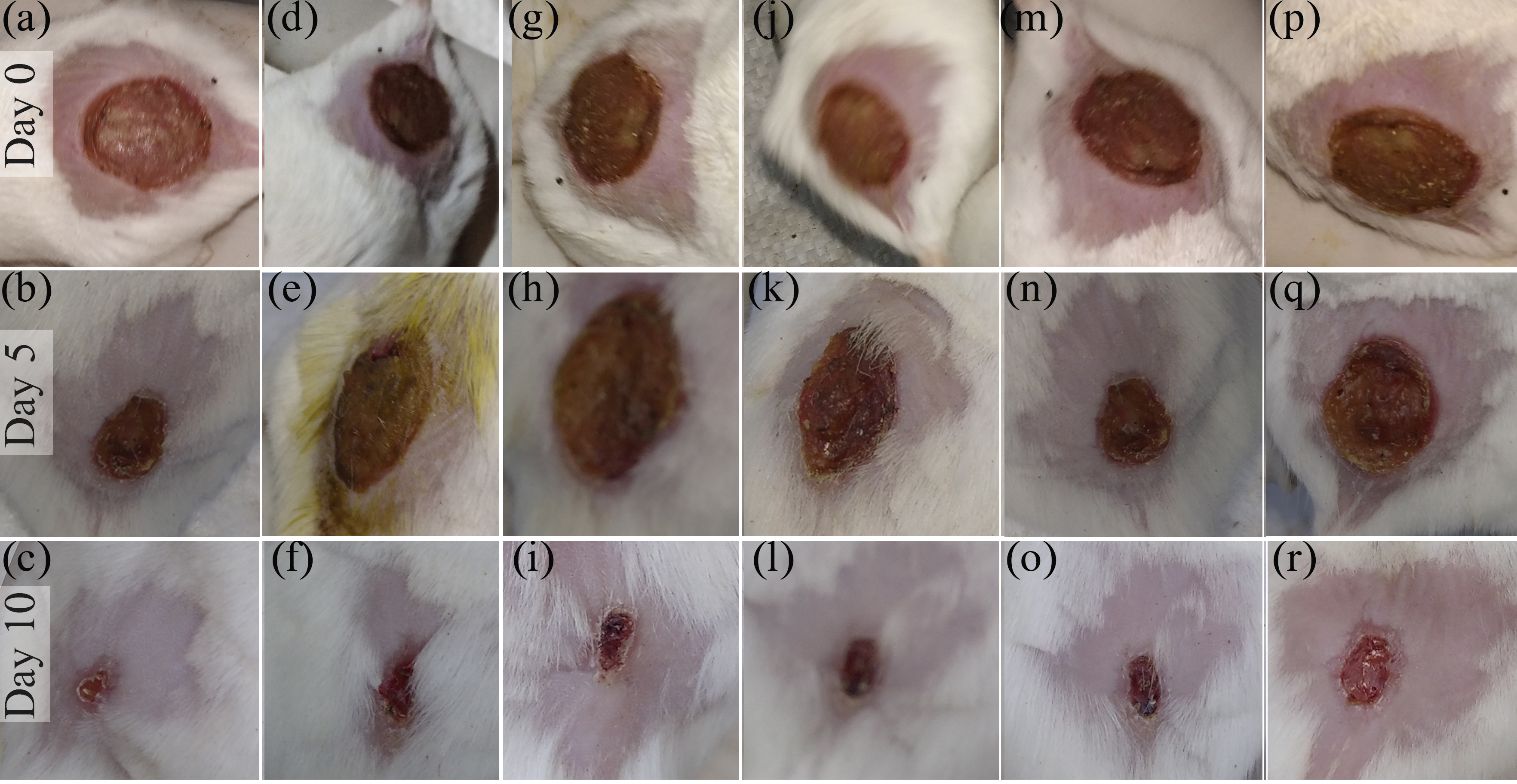

Supplement: Multimedia component 1 [file mmc1.zip › Figures all/Figure 1 day 0 5 10.jpg]

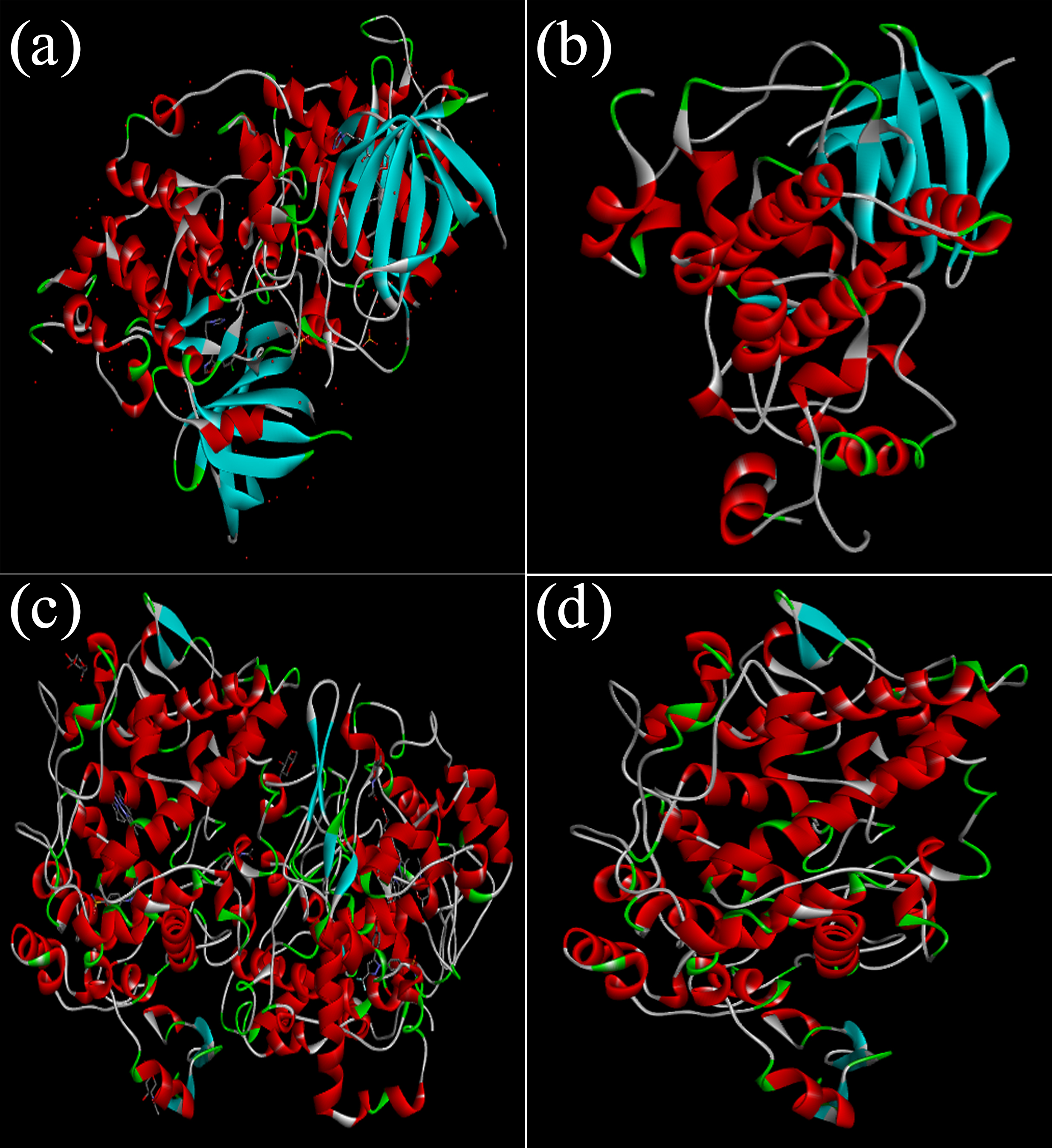

Supplement: Multimedia component 1 [file mmc1.zip › Figures all/Figure 2.jpg]

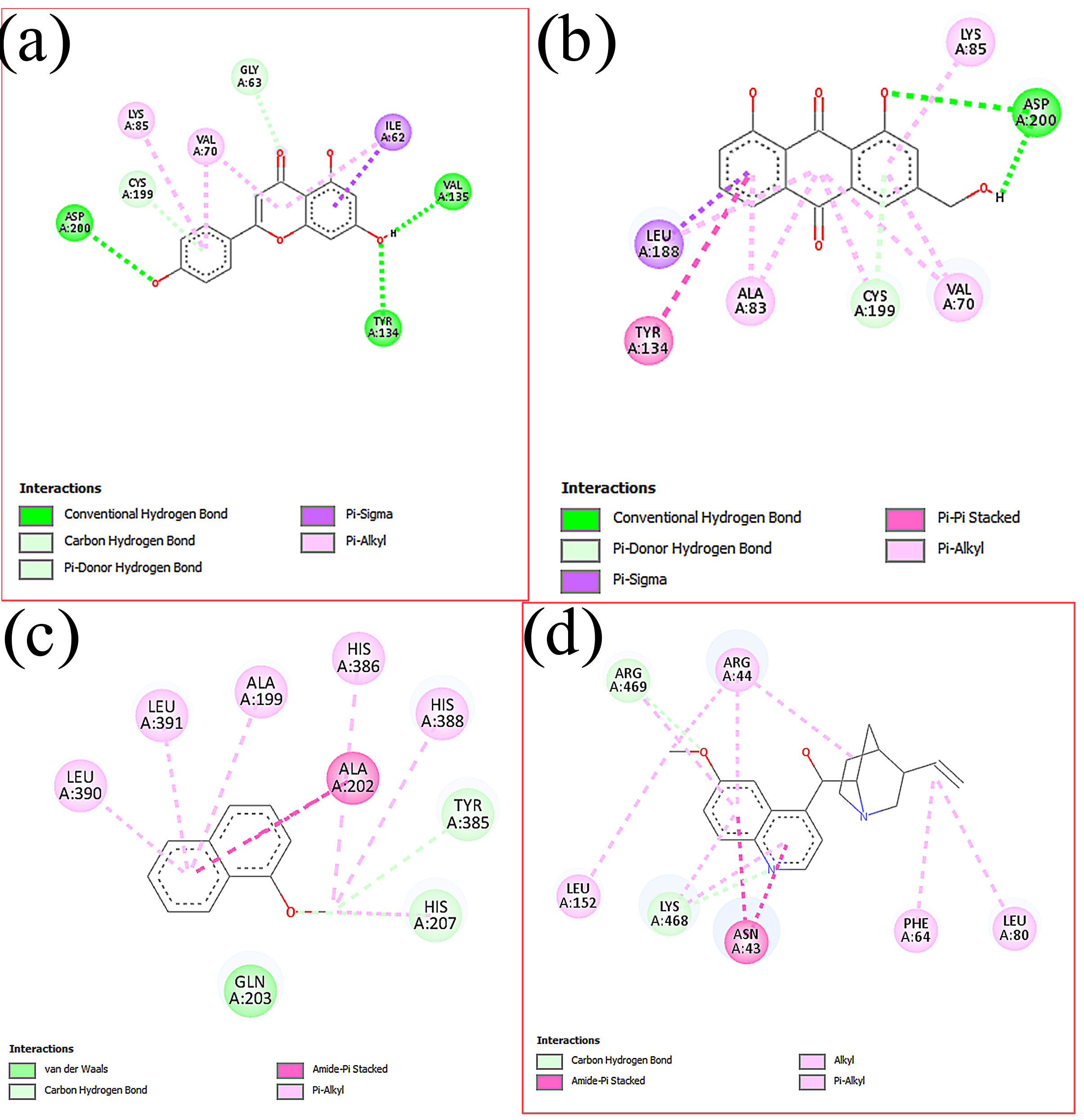

Supplement: Multimedia component 1 [file mmc1.zip › Figures all/Figure 3.jpg]

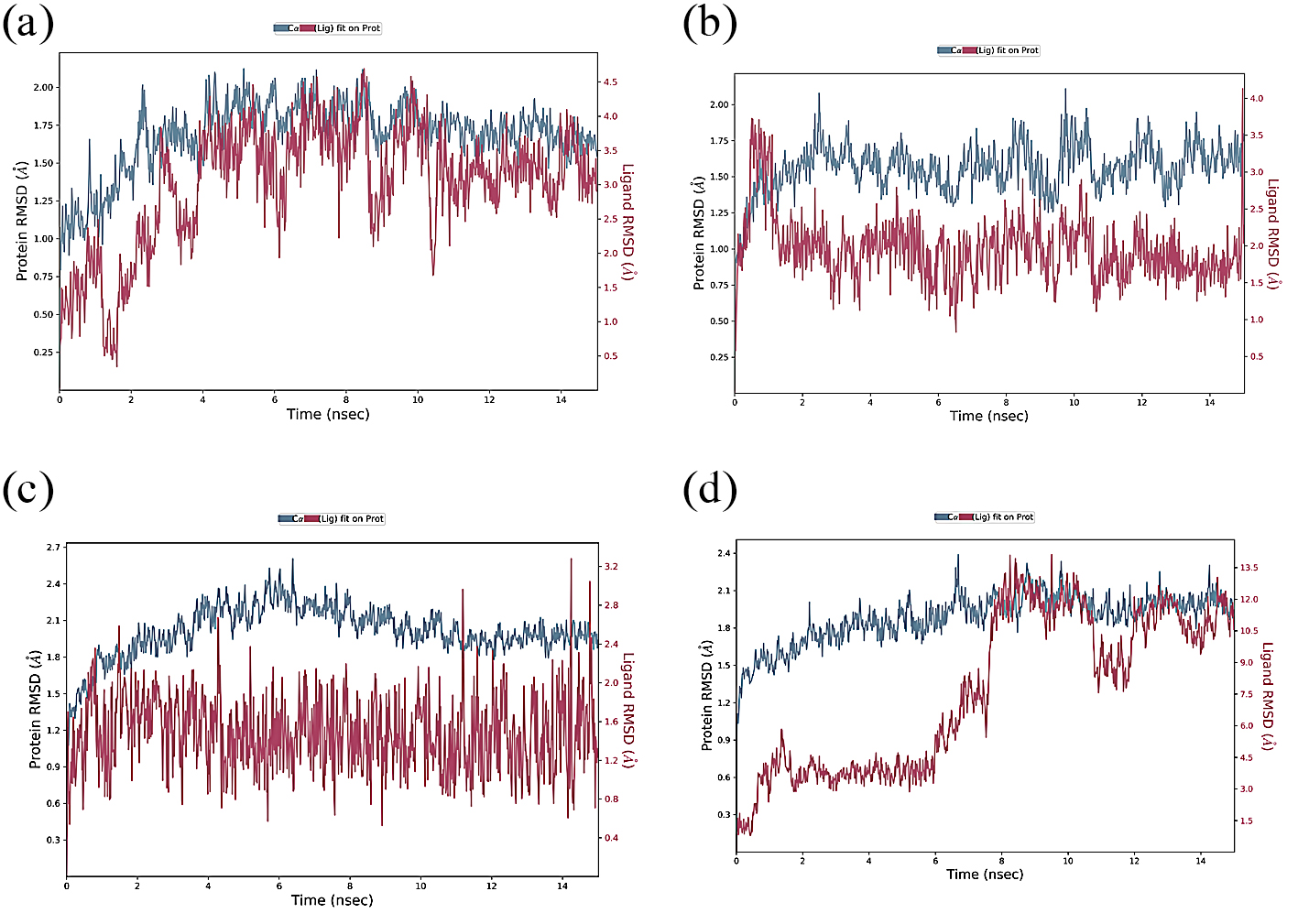

Supplement: Multimedia component 1 [file mmc1.zip › Figures all/Figure 4.jpg]

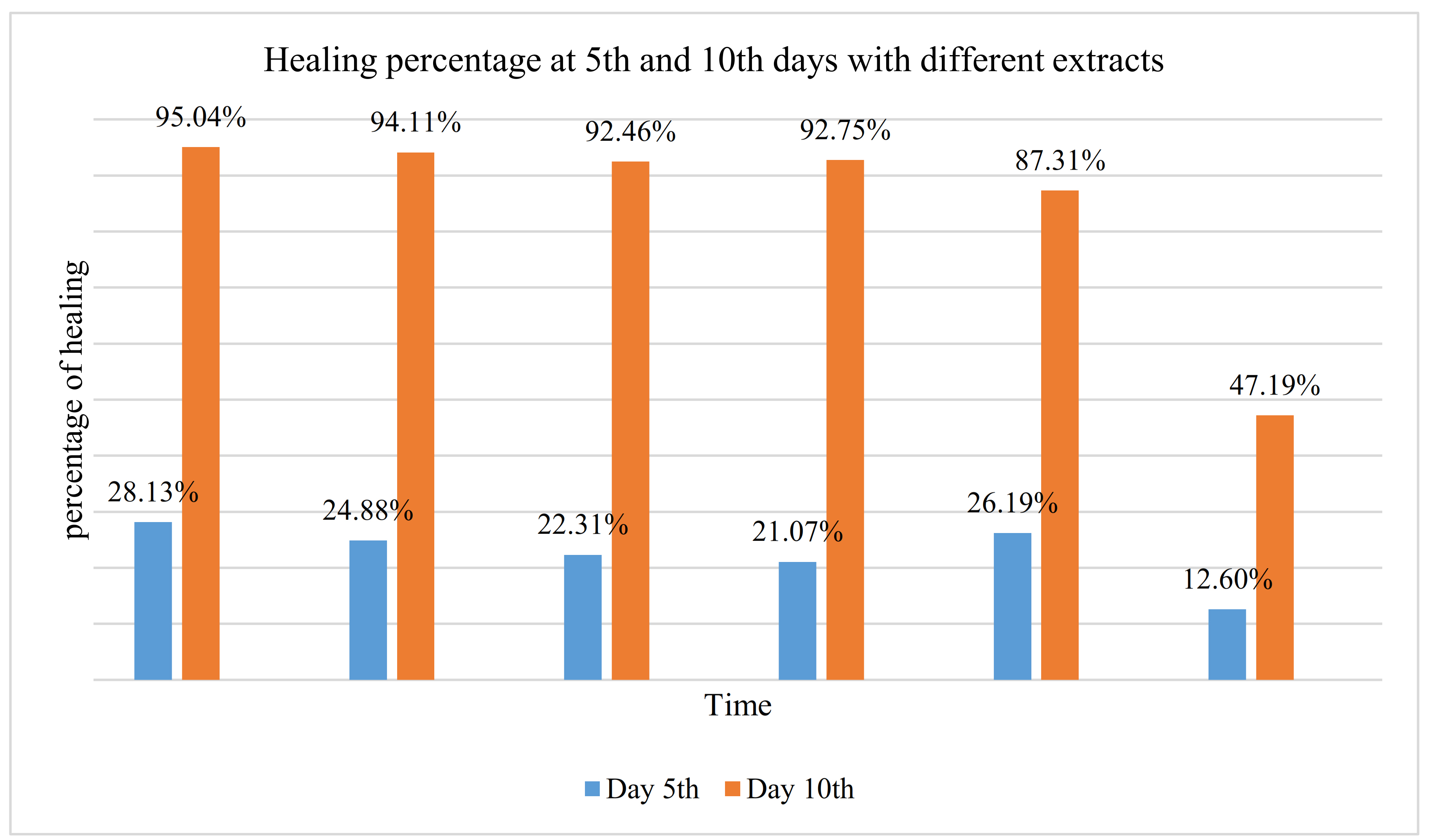

Supplement: Multimedia component 1 [file mmc1.zip › Figures all/Figure 5.jpg]

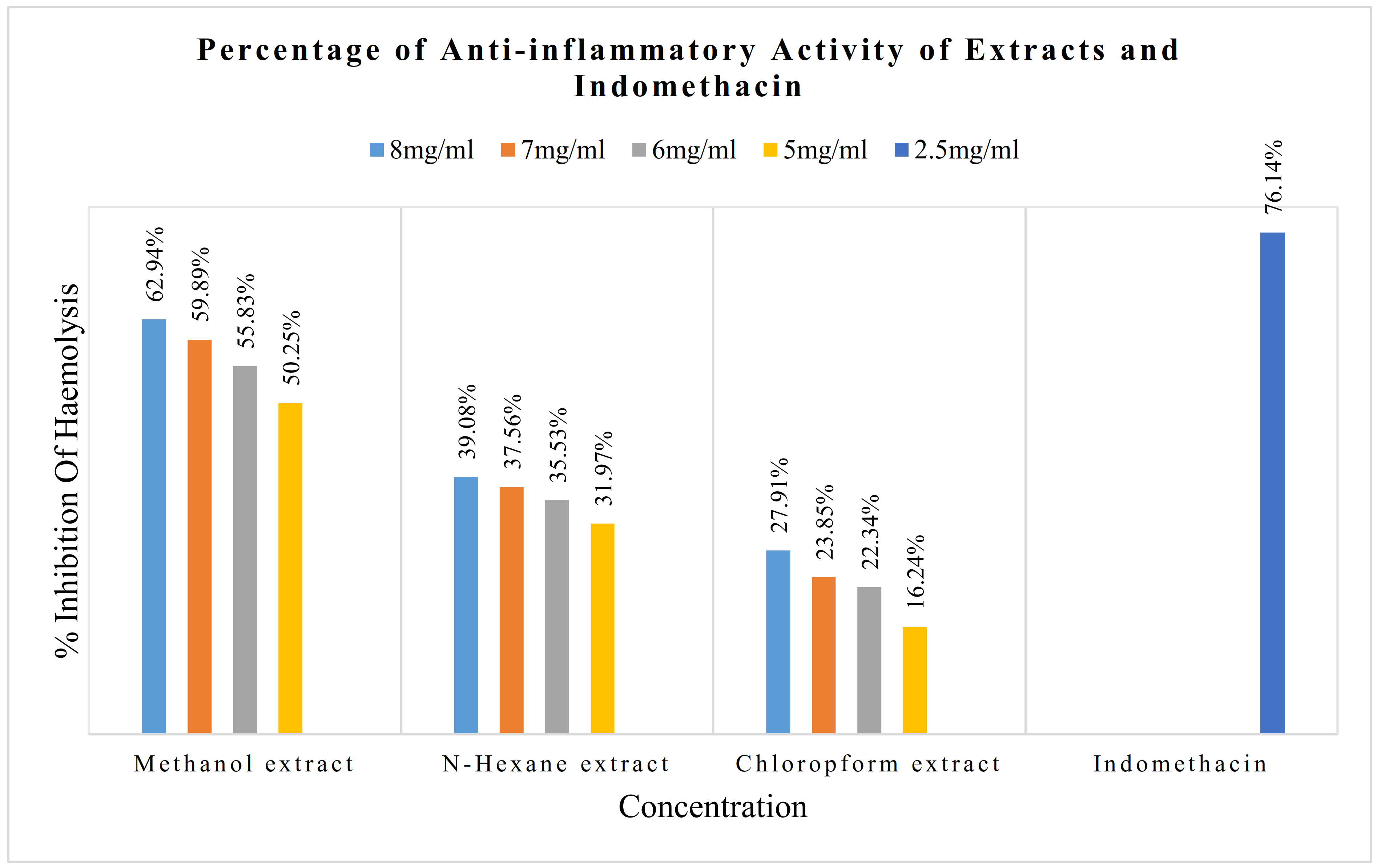

Supplement: Multimedia component 1 [file mmc1.zip › Figures all/Figure 6.jpg]
